# Media on‐demand: Continuous reconstitution of a chemically defined media directly from solids

**DOI:** 10.1002/bit.27738

**Published:** 2021-03-25

**Authors:** Daniel Komuczki, Gregory Dutra, Christoph Gstöttner, Elena Dominguez‐Vega, Alois Jungbauer, Peter Satzer

**Affiliations:** ^1^ Department of Biotechnology, Institute of Bioprocess Science and Engineering University of Natural Resources and Life Sciences Vienna Austria; ^2^ Center for Proteomics and Metabolomics Leiden University Medical Center Leiden Netherlands; ^3^ Austrian Centre of Industrial Biotechnology Vienna Austria

**Keywords:** CDM, CHO, continuous, IgG, solid

## Abstract

Chemically defined media are reconstituted batchwise and stored in hold tanks until use. To avoid large hold tanks and batchwise production of media, we developed continuous on‐demand reconstitutions directly from solids consisting of a hopper and a screw conveyor capable of feeding dry powdered media with the required precision ±5% at low dosing rates of 0.171 g min^−1^. A commercially available dry powdered cell culture medium was continuously fed over a duration of 12 h into a mixer which was connected to a UV‐cell for monitoring and the media were compared to a batchwise production. A comparable amino acid, carbohydrate, and osmolality profile to a batchwise reconstitution could be obtained. Cell cultivation showed comparable performance of batch and continuous reconstitution for two CHO cell lines producing the antibodies adalimumab and trastuzumab on a small and benchtop scale. In‐depth analysis of the produced antibodies showed the same glycosylation pattern, other posttranslational profiles such as methionine oxidation and deamidation compared to batchwise reconstitution. Therefore, we conclude a continuous reconstitution of the medium results in the same quality of the product. A continuous on‐demand media reconstitution will impact the supply chain and significantly reduce the floor space necessary for preparation and storage.

## Graphical abstract

In this study, we demonstrated a radical strategy for a continuous on‐demand reconstitution of a chemically defined media, which provides a solution for shrinking auxiliary buffer and media tanks necessary in continuous biomanufacturing. By using this strategy, we showed comparable performance and product quality of a CHO cell line expressing a monoclonal antibody.

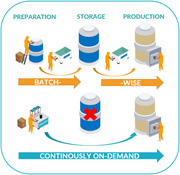

## 1 INTRODUCTION

The transformation from batch biomanufacturing to integrated continuous biomanufacturing leads to reduced capital costs and a footprint reduction of the individual unit operation (Arnold et al., 2019; Cataldo et al., 2020; Walther et al., 2015). However, a point often overlooked is that this transformation only “shrinks” the unit operations itself, while the necessary auxiliaries such as hold tanks, surge vessels, and demand for process materials are unchanged or even drastically increased. Hence, the supply chain is facing an increase in the demand for process materials and the necessity of handling significantly larger volumes. Correspondingly, companies optimize their supply chains, buying compounds in a solid form. That leads to an increased shelf life to avoid stock‐outs but, ultimately, it leads to increased storage costs. This is especially true for unit operations in continuous upstream processes. Particularly, if operated in a perfusion mode, several vessel volume exchanges per day of process materials such as cell culture media are required to yield economic competitiveness towards conventional fed‐batch processes (Cataldo et al., 2020). Harmonizing this transformation would either require outsourcing of the preparation procedure or higher efforts of a repeated just‐in‐time preparation of such cell culture media. Either way, both approaches would significantly contribute to the cost of goods (Gibson et al., 2019). A repeated reconstitution just in time ultimately leads to increased storage costs and could potentially lead to variations by human error or lot‐to‐lot variation in the process. This is especially crucial for the reconstitution of chemically defined media (CDM), which form the nutritional basis of cellular growth, productivity as well as the quality attribute of the protein of interest (POI). All ingredients of a CDM are by definition free of animal origin and depending on the organism they consist of a variety of components. For the cultivation of Chinese hamster ovary (CHO) cells, CDM supplies are growth factors, salts, vitamins, fatty acids, trace element, and amino acids (Eagle, 1959; Ham, 1965; Landauer, 2014; Salazar, Keusgen, et al., 2016; van der Valk et al., 2010). Imbalanced supply of nutrients has been shown to have severe effects on the culture performance and even product quality (Carrillo‐Cocom et al., 2015; Crowell et al., 2007; Duarte et al., 2014; Fomina‐Yadlin et al., 2014; Gramer, 2014; Guo et al., 2010; Kilberg et al., 2005, 2009; McCracken et al., 2014). Due to unavailable technology as well as concerns about the produced media composition, the media preparation for continuous processes is nowadays done in batch mode, requiring extensive cold storage capacity for large volumes of media which does not exist in all fed‐batch facilities. Additionally, the floor space for shuttling such amounts of media is not always available, which hinders equipping existing fed‐batch facilities with perfusion technology (Lin et al., 2017). Moreover, hold tanks limit the process development in terms of cost, footprint, and missing flexibility during the continuous process. For that reason, we developed a prototype at lab scale, which consists of a solid dispenser combined with a CSTR and a tubular reactor for continuous media or buffer preparation directly and continuously from solids. The advantage of an on‐demand reconstitution of the media continuously and directly from solids is that the footprint of the media preparation can be reduced drastically, saving on necessary tank storage. Also, by having the opportunity to reconstitute media continuously, new types of media formulations and feeding strategies might be enabled potentially leading to superior processes and consequently to superior product quality. For example, a gradual increase of nutrients in continuous processing can be achieved by changing the media composition, and therefore limitations in terms of solubility, availability but also stability can be prevented. Therefore, as the first proof of concept, we reconstituted commercially available CDM batch‐wise and continuously and compared the process performance using two CHO cell lines producing two different antibodies.

## 2 MATERIALS AND METHODS

All chemicals were of analytical grade and purchased from Sigma‐Aldrich, unless stated otherwise.

### 2.1 Experimental set‐up

**Figure 1:**
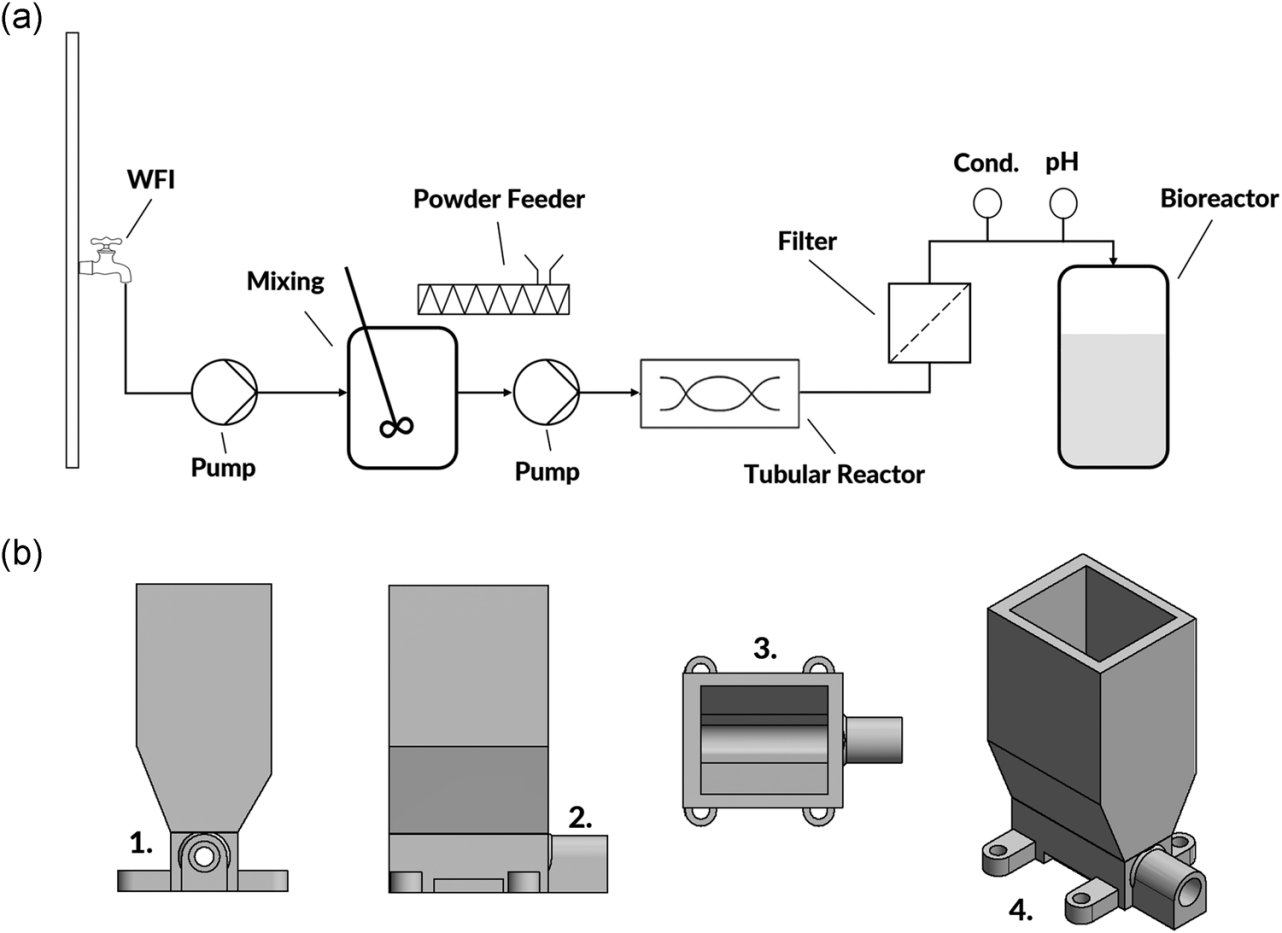
(a) Principle of the continuous on‐demand reconstitution of a chemically defined media. (b) The powder feeder with its (1) front, (2) side, (3) top view, and (4) 3D view

The prototype device at lab scale consisted of a 3D printed feeding hopper containing dry powdered media (DPM) and a screw conveyor. The DPM was fed by the screw conveyor into a mixing vessel, which is connected to an RO‐water infeed and the outflow is connected to a tubular reactor to complete mixing. The principle of the mode of operation is depicted in Figure 1. The device for feeding the DPM connected to a stepper motor had a dimension of 19 × 9 × 5 cm and the manufacturing steps as well as hardware and control were similar to a device used for the generation of in‐situ gradients. In short, a stepper motor controlling the screw conveyor and the device itself was mounted on a customized plate and installed in a closed box with slight overpressure. By changing the motion of the stepper motor, the feeding rate of the screw conveyor was controlled. The DPM which was stored in the hopper on the top of the screw conveyor ensured constant supply. For the control, we used a simple custom build python script. As the system was originally designed for buffer species, we adapted and redesigned the device to compensate for the difference in the flow behavior of DPM. The DPM was put into the hopper of the feeding device and calibration experiments were conducted for the commercially available chemically defined media Dynamis™ AGT™ (Thermo Fisher Scientific). The calibration experiments were performed using an Entris®Precision balance (Sartorius) connected to a Raspberry Pi 3 for data acquisition. Osmolality was confirmed using an OsmoTech® single sample osmometer (Advanced Instruments). The data were collected on‐line using the Simple Data Logger software (Smartlux SARL). For the continuous on‐demand reconstitution, the solids were fed into a miniaturized continuously stirred tank reactor (CSTR) with a magnetic stirrer and bottom outlet. The media was reconstituted according to the manufacturer's recommendation and a residence time (RT) in the continuous reconstitution according to the recommended mixing time.

In specific, the RT for short‐term reconstitution in the CSTR was 5 and 35 min for the tubular reactor. The medium powder was fed from the top by a screw conveyor at an average dosing rate of 0.202 g min^−1^ (±0.02; Figure 1). After the reconstitution, the medium was in‐line sterile filtered using 0.2 µm rapid‐flow filters (Thermo Fisher Scientific). We observed in the short‐term reconstitution that in the CSTR individual particles of the DPM were sometimes not completely dissolved. On the other hand, the medium had visually a comparable color after approximately an RT 18 min in the tubular reactor. Therefore, we slightly increased the RT in the CSTR to 6.5 min while shortening the RT in the tubular reactor to 22 min. After the reconstitution, the medium was sterile filtered using 0.2 µm rapid‐flow filters (Thermo Fisher Scientific).

### 2.2 Cell culture experiments

#### 2.2.1 Cell line and media

A CHO‐K1 expressing an Adalimumab derivate and a CHO‐S cell line expressing Trastuzumab both subtypes of immunoglobulin G1 (IgG1) were used. For pre‐cultures, the commercially available medium Dynamis™ AGT™ (Thermo Fisher Scientific) supplemented with 4 mM l‐Glutamine (Gln) and 1% anti‐clumping reagent was used. For the N‐stage we reconstituted continuously and batchwise Dynamis™ AGT™ (Thermo Fisher Scientific). Gln was added separately after the reconstitution and before media equilibration to an initial concentration of 4 mM, respectively. After thawing, cells were washed with freshly prepared basal medium at 180 g (5 min, 23°C). The supernatant was discarded, and cells were resuspended in fresh basal medium supplemented with 1% G418 and 4 mM Gln. All cell lines were grown in 125, 250, and 500 ml Erlenmeyer shake flasks. Cell cultures were grown at 36.5°C, 5% CO_2_, 70% humidity at 200 rpm in a MaxQ 2000 CO_2_ (Thermo Fisher Scientific) 19 mm orbital shaker and passaged in a 3‐day rhythm for four consecutive passages.

#### 2.2.2 Spin‐tube experiments

On the day of inoculation, cells were centrifuged at 180 g (5 min, 23°C). The fed‐batch experiments were carried out using Spin Tubes (TubeSpin®Bioreactor 50, TPP). The working volume (wv) was set at 15 ml and experiments were carried out in biological quadruplets, respectively. The media was equilibrated at process conditions for 2 h at 36.5°C. For all fed‐batch experiments, cells were inoculated at a seeding cell density (SCD) of 7 × 10^5^ cells mL^−1^ with a 1:10 dilution of the spent media in fresh media. Glucose levels were maintained above 3 g L^−1^ by daily bolus addition of a concentrated glucose stock solution (200 g L^−1^), if the glucose concentration was < 3 g L^−1^. The orbital shaker speed was set to 300 and to prevent masking effects no concentrated feed medium was introduced.

#### 2.2.3 Bioreactor experiments

On the day of inoculation, cells were centrifuged at 180 g (7 min, 23°C). For the bioreactor experiments, a DASGIP® Parallel bioreactor system was used (Eppendorf). The dissolved oxygen level was set at 50% and pH was controlled at 7.0 by gassing in of CO_2_ using the DASGIP® modules and addition of sodium bicarbonate. Working volume was set at 0.7 L, respectively. Bioreactor experiments were carried out in biological duplicates. The media was equilibrated at process conditions for 6 h at 36.5°C and stirrer speed was set at 150 rpm. For all fed‐batch experiments, cells were inoculated at an SCD of 6.5 × 10^5^ cells mL^−1^ with a 1:10 dilution of the spent media in fresh media. For the bioreactor experiments, glucose levels were maintained above 4 g L^−1^ by daily bolus addition of a concentrated glucose stock solution (200 g L^−1^), if the glucose concentration was <2 g L^−1^. Notably, to prevent masking effects no concentrated feed medium was introduced. A 1% solution of Antifoam C emulsion was added on demand.

#### 2.2.4 Process analytics

Samples were drawn daily from fed‐batch cultures for analysis of cell viability, glucose, and metabolite concentrations. Product concentration was determined at end of the cultivation on Day 7 (Spin Tubes) and Day 9 (Bioreactors), respectively. Cell concentrations and viabilities were measured during pre‐cultures and batch experiments using a Vi‐Cell™ (Beckman‐Coulter) using the trypan blue exclusion method. Daily glucose levels were analyzed by the blood glucose monitoring system Contour X (Bayer). Osmolality was measured using the OsmoTECH® single‐sample micro‐osmometer (Advanced Instruments). Ammonium hydroxide levels were measured by a Cedex BioAnalyzer (Roche). Samples were sent to a laboratory for carbohydrate and amino acid quantification.

### 2.3 Protein purification

The antibodies were captured using preparative protein A chromatography with a conventional chromatographic workstation ÄKTA Pure 25 coupled with a fractionating module (Cytiva). The equilibration, wash 1, wash 2, and elution buffers were 50 mM phosphate buffer, pH 7.4; 50 mM phosphate buffer, pH 5.0; 50 mM phosphate buffer and 1 M NaCl, pH 7.4 as well as 100 mM Glycine buffer pH 3.5. Protein A MabSelect Sure resin (Cytiva) was packed in a HiScale 26 housing (Cytiva) resulting in a column volume of 24.4 ml. For purification, the column was equilibrated with 10 CV equilibration buffer and loaded with 450 ml of culture supernatant. Washes were performed with 10 CV of wash buffer 1, and 10 CV of wash buffer 2. The product was eluted with a linear gradient of 5 CV to the elution buffer and an additional 5 CV of elution buffer. The sample was collected using the fractionating system connected to the ÄKTA and was neutralized to pH 7. The process was monitored by the conductivity, pH, and absorbance at 280 nm through the Unicorn 7.0 Software (Cytiva).

### 2.4 HPLC protein A chromatography

HPLC protein A affinity chromatography was used to determine the antibody concentration. A Dionex UltiMate 3000 HPLC system was equipped with a diode array detector (Thermo Fisher Scientific). Mobile phase A was a 50 mM phosphate buffer, pH 7.0. Mobile phase B was a 100 mM glycine buffer, pH 2.5. Before usage, all buffers were filtered through 0.22‐μm filters (Merck KGaA) and degassed. The system was run at a flow rate of 2.5 mL min^−1^. We loaded 20 μl of the sample, filtered, on a POROS A 20‐μm column (2.1 × 30 mm^2^, 0.1 ml; Thermo Fisher Scientific). The column was equilibrated with 10 column volumes of mobile phase A, eluted with a step gradient with 20 column volumes of 100% mobile phase B, and re‐equilibrated with 30 column volumes of mobile phase A. The absorbance at 280 nm was measured. We used a similar protein A purified IgG1 as the calibration standard. The calibration range was 0.1–8 mg mL^−1^. We evaluated and quantified the results with the Chromeleon™ 7 software (Thermo Fisher Scientific).

### 2.5 Size exclusion chromatography

To estimate product purity, size exclusion chromatography was performed. For HPLC analytics, we used a Dionex UltiMate 3000 HPLC system equipped with a diode array detector (Thermo Fisher Scientific). The running buffer was a 50 mM potassium phosphate buffer with 150 mM NaCl, pH 7.0 (Merck KGaA). The buffer was filtered through 0.22‐μm filters (Merck KGaA) and degassed. We applied 20 μl of the filtered sample to a TSKgel® G3000SWXL HPLC column (5 μm, 7.8 × 300 mm^2^) in combination with a TSKgel SWXL Guard column (7 μm, 6.0 × 40 mm^2^; Tosoh). The absorbance at 280 nm was recorded, and the results were evaluated with the Chromeleon™ 7 software (Thermo Fisher Scientific). The antibody purity was calculated as the ratio of the monomer peak area (retention time: 21.2 min) to the sum of all peak areas, based on the 280 nm signal.

### 2.6 Intact protein analysis by sheathless CE‐MS

For intact mass analysis, the antibodies were buffer exchanged (three cycles) to 10 mM ammonium acetate (AmAc) pH 3.1 using 10 kDa Vivaspin MWCO (Sartorius) at 10,000*g* at 4°C. Consequently, the antibodies were adjusted to a final concentration of 1 μg/μl with 50 mM AmAc pH 3.1. Intact antibody samples were analyzed using a Sciex CESI 8000 instrument coupled via an XYZ stage to an Impact qTOF‐MS (Bruker Daltonics). Bare fused silica capillaries containing a porous tip (Sciex) were used for separations. Prior to analysis, the capillary was coated using a polyethyleneimine coating and evaluated using a protein test mixture following the protocol reported by Sciex (Santos et al., 2015). For the separation of the mAbs, a background electrolyte (BGE) consisting of 10% acetic acid was used. Before each run, the capillary and the conductive line were flushed with BGE for 2 min (100 psi, forward pressure) and 2 min (75 psi, reverse pressure). The injection time of the mAbs was 15 s (2.5 psi), followed by a plug of BGE for 25 s (0.5 psi). The samples were separated at 20 kV (reversed polarity) and a temperature of 20°C for 45 min, followed by a ramp down to 1 kV in 5 min. For mass spectrometric detection, the system was operated in positive ion mode. The capillary voltage was set at 1200 V, and the temperature and flow of the dry gas were 180°C and 1.8 L/min, respectively. The collision cell voltage was set to 20 eV and the quadrupole ion energy to 5 eV. To achieve proper declustering of the antibodies 100 eV were used for in‐source collision‐induced dissociation. The pre‐pulse storage time was set to 25 μs and the transfer time to 120 μs. For the analysis, the *m/z* range between 500 and 6000 was monitored. Data analysis and deconvolution were performed using the DataAnalysis software from Burker Daltonics.

## 3 RESULTS AND DISCUSSION

**Figure 2:**
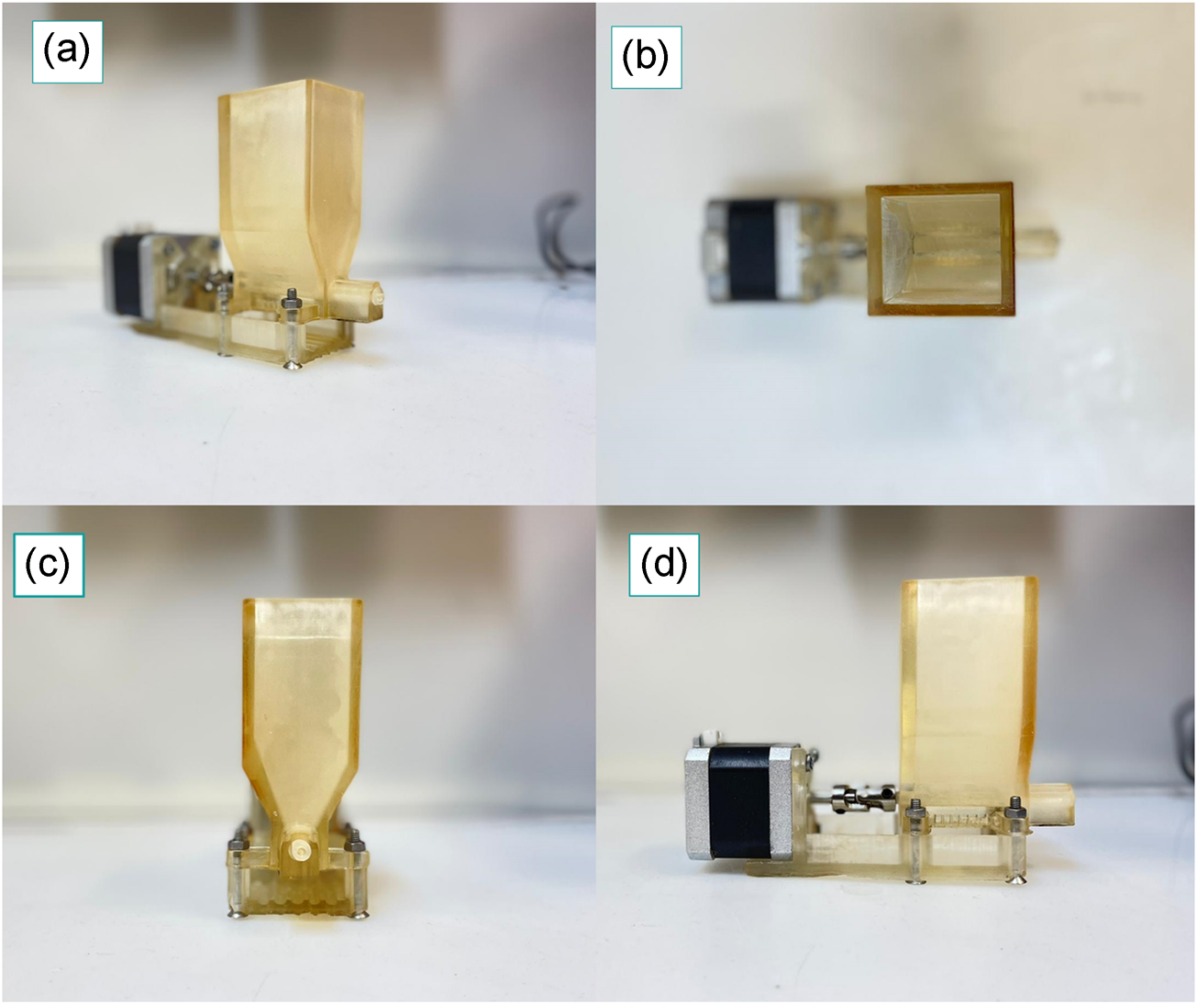
(a) The design of the device for the feeding of dry powdered medium with its (b) top‐, (c) front‐, (d) and side view [Color figure can be viewed at wileyonlinelibrary.com]

Despite the commercial availability of automated media and buffer preparation units, none of these systems solve the shortcomings of being able to reconstitute significant amounts of process materials continuously on‐demand at the point of use. Recently, we reported an in‐house developed device consisting of a screw conveyor, feeding hopper, and a control unit capable of generating in‐situ gradients for chromatographic separations by direct addition of solids (Komuczki et al., 2020). Thereby, solid buffer components were stored in the hopper for an on‐demand point of use generation of highly linear gradients for chromatography. In this study, we adapted the system for a continuous reconstitution of a CDM directly from solids. This continuous on‐demand reconstitution of CDM can substantially shrink auxiliary buffer and media tanks needed for continuous upstream production like perfusion systems. Undoubtedly, CDM differ significantly in their powder characteristics and flow behavior from buffer species commonly used in the biopharmaceutical industry simply due to their more complex formulations and manufacturing (Fike et al., 2001; Salazar, Bleifuß, et al., 2016). In contrast, most used buffers in the biopharmaceutical industry, are consisting of a few salts (Carredano et al., 2018). For that reason, we had to adapt our device for the differences in powder flow behavior by redesigning the geometrical shape, power translation, and screw design (Figure 2; Jenike, 1964; McGlinchey, 2009). More details on the manufacturing of the device can be found in a previous publication (Komuczki et al., 2020).

The core unit of our developed system is a 3D printed powder feeder, which continuously feeds DPM in a CSTR having an in‐ and outflow. We tested the device for accuracy, precision, and stability prior to the continuous reconstitution at different dosing rates. As can be seen from Figure S1, increased rpms decreased the accuracy of the dispensing, however, in the range of our selected dosing rates was below 5%. The error at higher rpms is caused by inhomogeneous 3D fabrication, which leads to friction during the power translation on the screw and therefore reduces the accuracy and precision of the feeding rate. Solving this inhomogeneous 3D fabrication would either require a more sophisticated 3D printer, which would allow a higher printing resolution or a scale‐up of the proposed device. We evaluated the continuous on‐demand reconstitution for the basal cell culture media Dynamis™ AGT™ in short‐ and long‐term experiments. The medium reconstituted in short term was used for the spin tube and long term for the bioreactor experiments. The reconstitution of the CDM Dynamis™ AGT™ requires no additional pH adjustments during the dissolution and therefore no in‐line pH‐adjustment was integrated in the system. Nevertheless, we think that applying the system to other media requiring pH‐adjustment is possible, as in‐line pH adjustment has been demonstrated successfully already at an industrial scale. Nevertheless, the system developed described herein is currently limited by not being a fully closed system and without any environmental control, which would be pivotal for an industrial application due to reasons for dust control, the risk of contamination, and potential degradation of components, but it would be out of the scope of the proof of concept (Matheson, 2005; Salazar, Bleifuß, et al., 2016; Sheraz et al., 2014). Monitoring of the degradation of dry powder media components during storage in the hopper could be conducted by colorimetry as recently shown by Dickens et al. (2020). Besides that, closed powder feeding units at a larger scale such as twin‐screw conveyors operating in a closed fashion are already commercially available, which would solve the addressed issues.

### 3.1 Short‐term reconstitution

For a first continuous on‐demand reconstitution of the CDM, we used a simplified set‐up without any on‐line monitoring and volume control. We ensured a constant volume in the CSTR (40.6 ml) for a total run time of 2.9 h by visual monitoring of a scale and synchronization of inlet and outlet pumps. Media powder was fed from the top by the device at a dosing rate of 0.202 g min^−1^ (±0.02; Figure S1). After a ramp‐up phase of 40 min, a continuously reconstituted medium was collected in 40 ml fractions. The lack of a control system for the volume in the CSTR led to a decrease of the volume in the CSTR after 40 min. Consequently, the feed rates of the pumps were manually adjusted to ensure a constant volume. After solving these issues, a relative constant volume (±6.5%) was achieved. For the biological tests, we pooled the aliquots showing constant osmolality and pH (Figure S2) neglecting aliquots corresponding to the noted process deviations. The pools were sterile filtered and used for the spin tube experiments.

**Figure 3:**
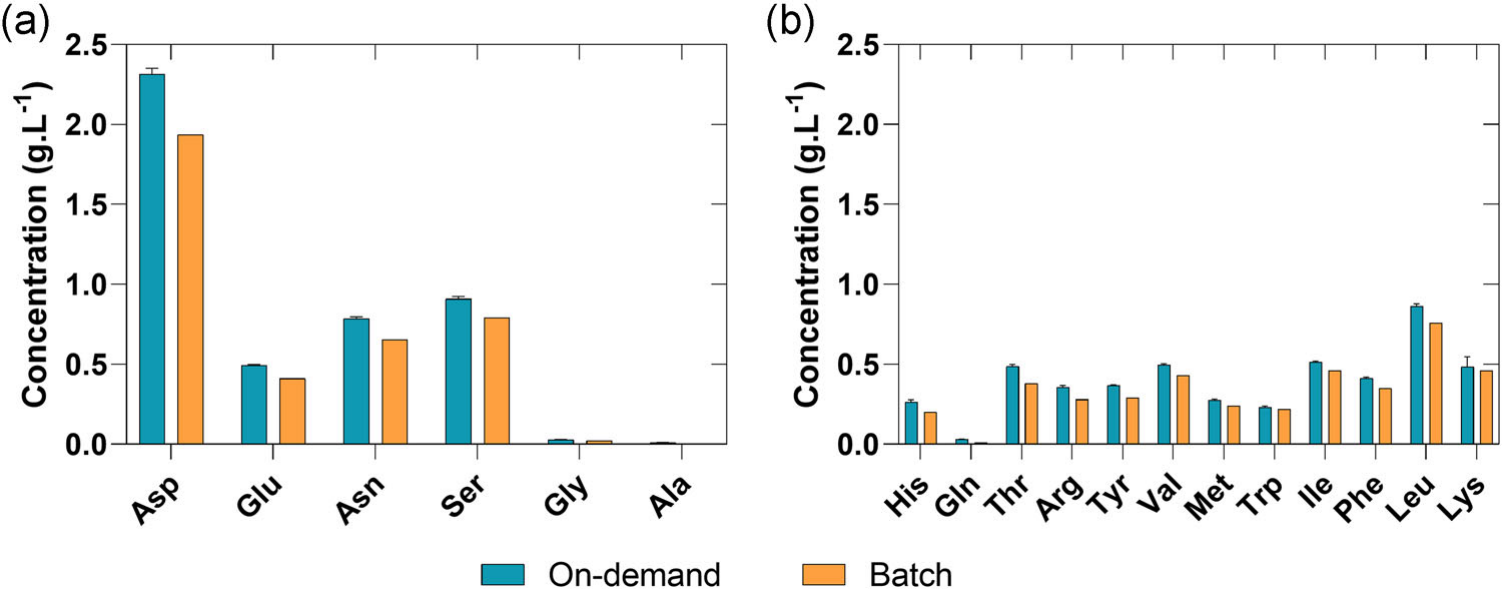
(a) Non‐essential and (b) essential amino acid concentration of the short‐term continuous on‐demand reconstitution and batch reconstitution [Color figure can be viewed at wileyonlinelibrary.com]

Analysis of the amino acid profiles of the different reconstitution modes indicates an overall slight concentration increase of amino acids (AA) for the continuous on‐demand short‐term reconstituted medium (Figure 3) in respect to non‐essential (NEAA – A) and essential (EAA – B) amino acids. This is most likely due to a minor mismatch of solid feed rate and liquid feed rate resulting in a slightly overconcentrated media. The amino acid distribution is the same for both reconstitution methods and an appropriate control strategy for solid and liquid flow rates will avoid such deviations entirely.

### 3.2 Cell culture performance – Spin tube

**Figure 4:**
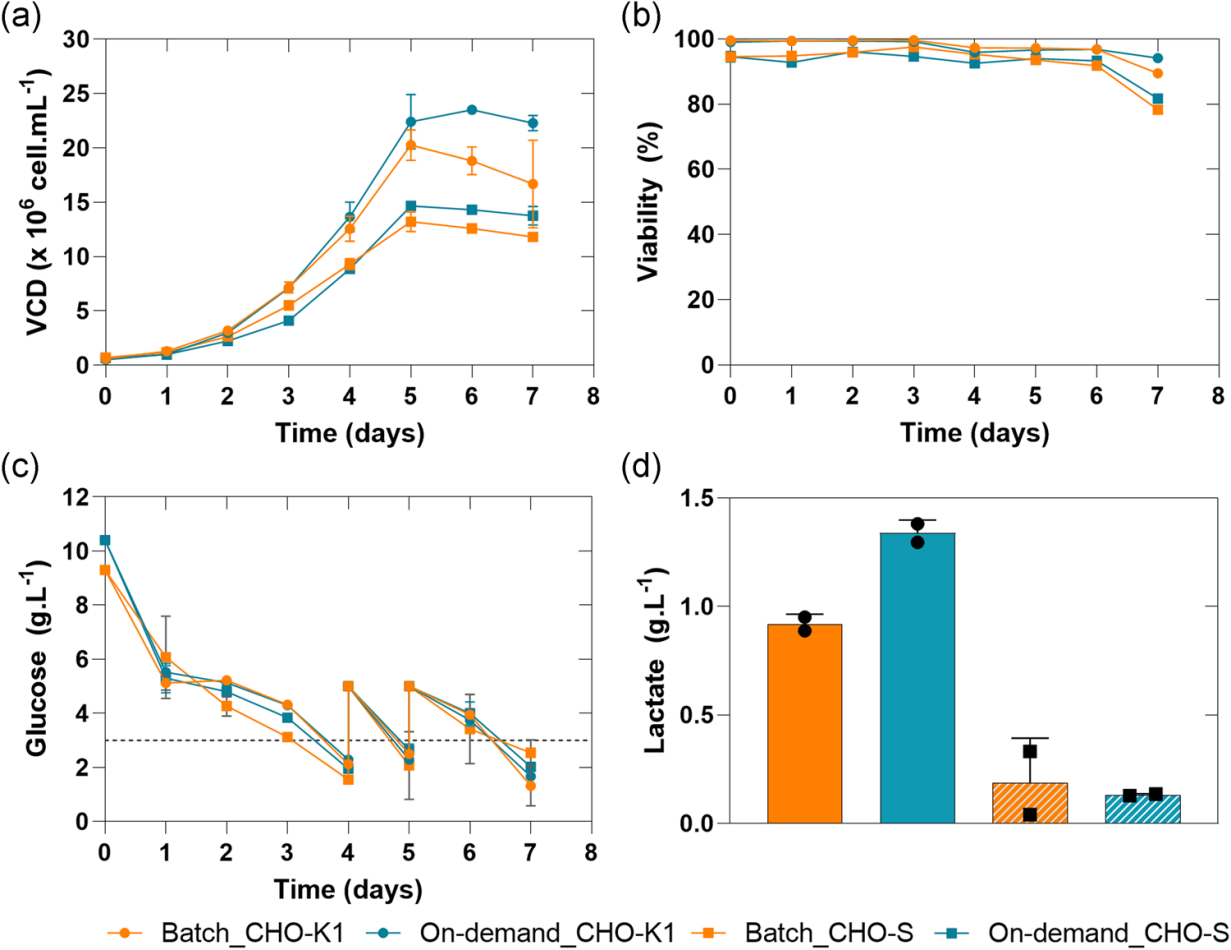
(a) Viable cell density, (b) viability (c) glucose and final lactate concentration profiles (d) of a CHO‐K1 and CHO‐S cell line in short‐term continuous on‐demand (*n* = 2) and batchwise reconstituted medium (*n* = 4) [Color figure can be viewed at wileyonlinelibrary.com]

The continuously reconstituted and batch reconstituted media were tested for their biological performance using a CHO‐S and a CHO‐K1 cell line in spin‐tube fed‐batch experiments producing different IgGs. Thereby, we evaluated the cell culture performance in terms of growth, metabolites, and productivity. Irrespective of the reconstitution mode of the CDM, both cell lines lead during the fed‐batch experiments to a comparable cell culture performance and metabolite profiles (Figure 4). For the CHO‐K1 cell line, this resulted in an average maximum viable cell density (MVCD) of 23.50 (±0.06) × 10^6^ cells mL^−1^ for the continuous on‐demand and 20.25 (±1.22) × 10^6^ cells mL^−1^ for the batch medium (Figure 4). However, the CHO‐S cell cultures of resulted for the batchwise reconstituted medium in 12.59 (±0.69) × 10^6^ cells mL^−1^ and for the continuous on‐demand to a 14.25 (±0.27) × 10^6^ cells mL^−1^. The CHO‐K1 cultures had at the day of harvest still viabilities above 89% and the CHO‐S cultures dropped below a viability of 82%, irrespective of the mode of reconstitution. Likewise, glucose was consumed at a similar rate which required bolus addition from Day 4 onwards for both cell lines. The lactate concentration at the day of harvest differed slightly for the CHO‐K1 and CHO‐S cultures. This slightly higher by‐product formation for the on‐demand cultures resulted most likely from the marginally higher VCD which was caused by the more concentrated on‐demand medium. Correspondingly, analysis of the final titer resulted for the CHO‐K1 in 0.27 (±0.001) g L^−1^ for the on‐demand and 0.22 (±0.08) g L^−1^ for the batch cultures. For the CHO‐S cultures analysis of the final titer resulted 0.20 (±0.001) g L^−1^ (on‐demand) and 0.18 (±0.001) g L^−1^ (batch), respectively.

As can be seen from Figure S3 (A,B) also the amino acid profiles at the last day of the cultivations show a relatively comparable amino acid profile within essential and non‐essential amino acids.

### 3.3 Long‐term reconstitution

**Figure 5:**
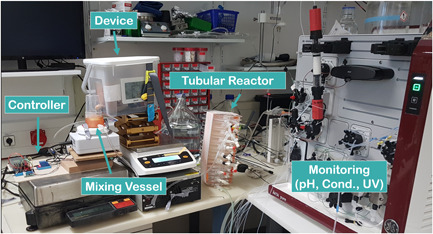
The experimental setup of the continuous on‐demand reconstitution of a CDM (long term). Thereby, the device feeds solid particles into a mixing vessel, which is consequently mixed in a tubular reactor. At the end of the tubular reactor, the now liquid medium is sterile filtered and either transported into a bioreactor or a hold tank. CDM, chemically defined media [Color figure can be viewed at wileyonlinelibrary.com]

As we saw limitations in the stability of the system using manually controlled pumps for the inlet and the outlet of the CSTR in the system, we reevaluated and adapted the system to be operational for a long‐term reconstitution. In detail, we build a simple control unit loop that ensured a constant working volume (45 ml) in the mixing vessel and integrated the device into a confinement experiencing slight over‐pressure with dry process air to prevent moisture accumulation (Figure 5). A peristaltic pump connected to an Arduino resupplied the stirring vessel with fresh RO‐water and one dual‐piston pump of the ÄKTA system set at 6.9 mL min^−1^ transported the medium through the tubular reactor as illustrated (Figure 1). Dry powdered medium was dosed at 0.171 g min^−1^. The flow path of the ÄKTA system was set to by‐pass for on‐line monitoring of pH, UV, and conductivity.

**Figure 6:**
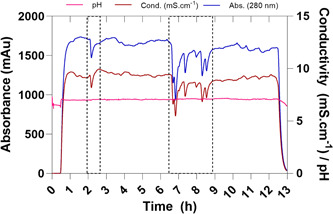
Monitoring of pH (pink), conductivity (dark red), and UV absorbance (280 nm, blue) of the continuous on‐demand reconstitution of a chemically defined media over a duration of 12 h. Red dashed boxes indicate the temporary failure of the system by arching and increase in relative humidity within the confinement [Color figure can be viewed at wileyonlinelibrary.com]

After a ramp‐up phase of 50 min, the continuous on‐demand reconstitution entered stable conditions illustrated by the signal measured for the UV, conductivity, and pH (Figure 6). A ramp‐up phase of 50 min might be problematic for certain industrial applications. However, by following a fast ramp‐up approach achieved by starting with an empty vessel, closing the outlet of the CSTR and normal infeed of liquid and solid, while only starting the operation once the vessel is filled to the volume intended for constant operation, the ramp‐up phase can be reasonably reduced to the RT of the CSTR. After 2 and 6 h (Figure 6, black dashed box) arching in the hopper and moisture accumulation, both common phenomena's in the handling of bulk solids, could be observed which lead to a temporary failure of the system (Jenike, 1964; McGlinchey, 2009). Insufficient supply of dry process air was likely the cause for the first deviation. However, failure of the sealing of the confinement probably leads to accumulation of moisture and to the second deviation. Nevertheless, manual removal of the arch and increasing the flow of the process air entering the confinement resolved the failure and restored the reconstitution to stable conditions. Thereby, we are able to continuously reconstitute a CDM on‐demand for a duration of 12 h. Notably, we observed that powder, which was not properly discharged by the screw, lead to clump formation and inhomogeneous discharge of the powder. Nevertheless, by establishing a closed system while simultaneously monitoring and controlling the dosing accuracy on‐line using an integrated scale the error introduced by the clump formation would be resolved. In fact, any fast fluctuation will be sufficiently smoothed out by the CSTR.

**Figure 7:**
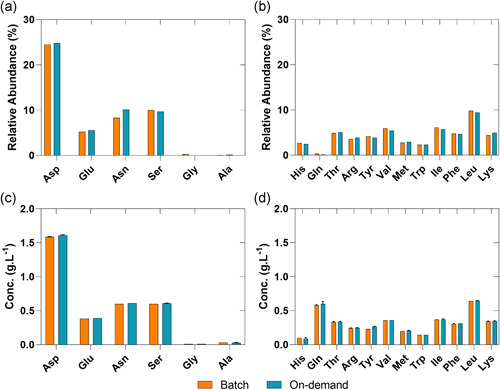
Relative abundance of individual amino acid of long‐term continuous on‐demand and batchwise reconstituted medium [Color figure can be viewed at wileyonlinelibrary.com]

The inhomogeneous powder discharge and probably slight fluctuations in the volume of the CSTR with different dissolution kinetics of the individual components are most likely the reason for the fluctuations observed in the stable parts for the conductivity (±3.27%) and UV signal (±2.97%). Analysis of the dosing accuracy and precision as a function of weight over the duration of 12 h showed that the device, besides the two deviations, was within the expected accuracy for such small feeding rates (±5%) with a slightly lower but accurate feeding rate of 0.162 g min^−1^ in contrast to the anticipated feeding rate of 0.171 g min^−1^ (−5%; Figure S3). The robustness of the device is further demonstrated over the duration of the feeding of powder by tracking the fed amount of CDM by weight (Figure S4). However, we suggest additionally implementing on‐line monitoring of weight and a control loop for the feeding of CDM as well as a control loop for the feeding of liquid into the system. Measurement of the osmolality for the on‐demand collected medium during the stable part of the reconstitution resulted in 276 mOsm Kg^−1^ and 280 mOsm Kg^−1^. However, analysis of the conventional reconstituted medium resulted in a difference of 7% resulting in 296 mOsm Kg^−1^ which is undoubtedly caused by the slightly lower feeding rate detected for the continuous reconstitution. Thus, to ensure the same starting conditions we diluted (+7%) the batchwise reconstituted media to the same osmolality using RO water. To highlight any inconsistencies that might arise due to inhomogeneous dissolution of the CDM in comparison to the batchwise reconstitution (e.g., nonuniform amino‐acid dissolution leaving one or more components as solids), we analyzed the amino acid composition after the reconstitution and at the day of inoculation of the continuous on‐demand reconstituted media (Figure 7A,B).

Notably, an insufficient supply of nutrients could reduce the maximum possible yield, cellular growth, and performance, alter the amino acid sequence of the POI or even trigger metabolic response pathways which ultimately lead to a growth arrest (Altamirano et al., 2004; Carrillo‐Cocom et al., 2015; Fomina‐Yadlin et al., 2014; Gramer, 2014; Guo et al., 2010; Kilberg et al., 2005, 2009; Popp et al., 2015; Xing et al., 2011). As illustrated in Figure 7 the relative abundance (A, B) of the individual amino acids and concentrations at the day of inoculation (C, D) after the continuous on‐demand show a comparable profile to a conventional reconstituted media.

### 3.4 Cell culture performance – Bioreactor

**Figure 8:**
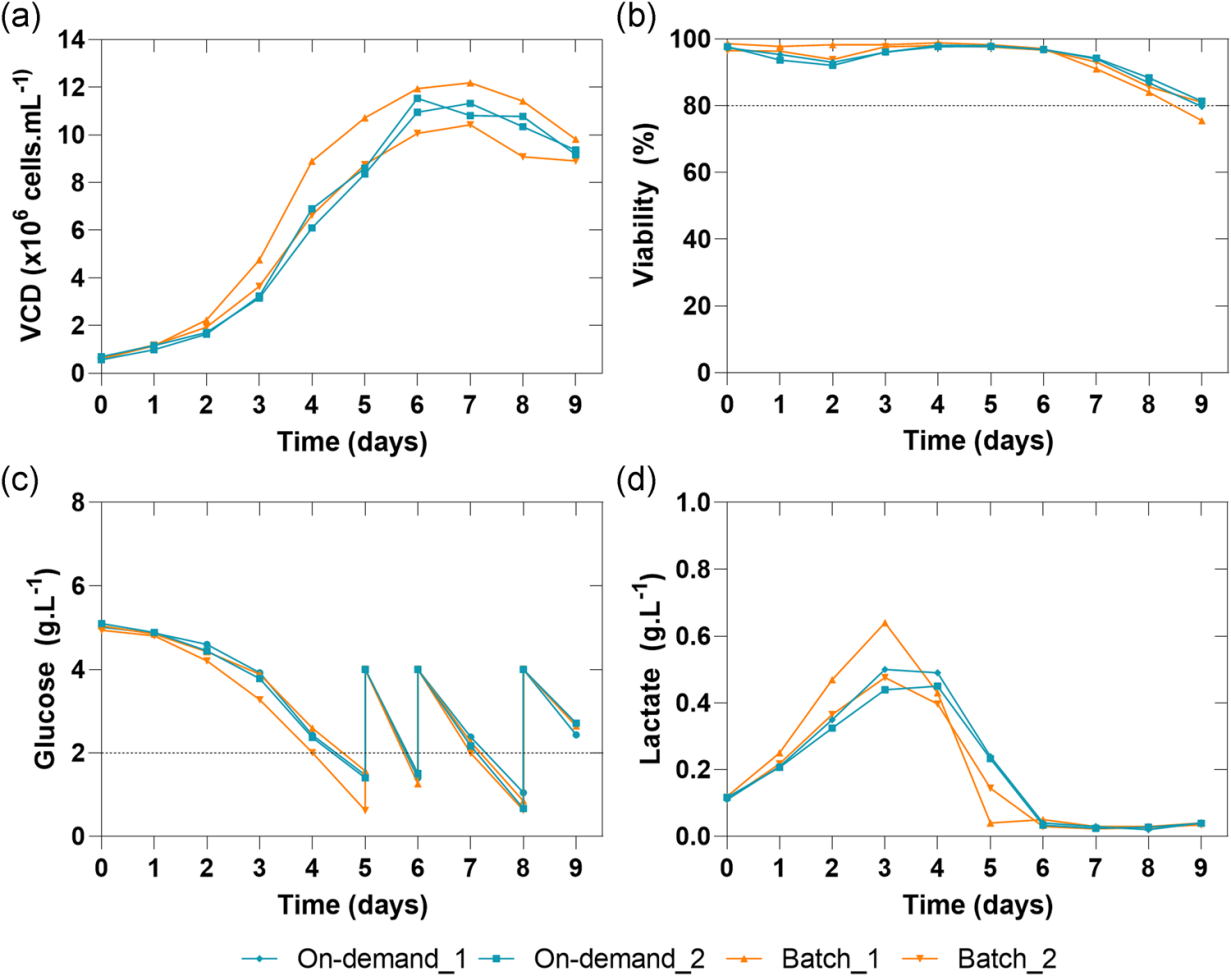
(a) Viable cell density, (b) viability, (c) glucose, and (d) lactate profiles (*n* = 2) of the batchwise and continuous on‐demand reconstituted medium of the bioreactor fermentations [Color figure can be viewed at wileyonlinelibrary.com]

With material pooled from long‐term continuous reconstitution, we performed an additional cell culture experiment on a larger scale in controlled bioreactor conditions to study potential differences between the long‐term continuous on‐demand and batchwise reconstituted medium. For the experiment, we used the CHO‐K1 cell line in controlled conditions using DASGIP bioreactors (*n* = 2). Likewise, to the spin‐tube experiments, both approaches of medium reconstitution lead to the same performance (Figure 8). The pooled medium collection (On‐demand_1, On‐demand_2) of continuous on‐demand reconstituted medium lead to an MVCD of 11.53 and 11.32 × 10^6^ cells mL^−1^ and the cultures grown in batchwise reconstituted medium reached an MVCD of 12.19 and 10.43 × 106 cells mL^−1^, respectively (Figure 8). Although the overall growth profile was shown to be comparable, we observed a shortened lag‐phase for the Batch_1 culture which resulted in a steeper growth during the exponential phase. Nevertheless, the difference at the beginning of the fermentation diminished over the duration of the fermentation.

This is further supported by the viability of the cultures, which lead at the day of the harvest for the On‐demand_1 and On‐demand_2 cultures to the final viability of 79.80% and 81.40%. However, the Batch_1 and Batch_2 cultures lead to the final viability of 75.50% and 81%, respectively. A similar trend could be observed for the glucose, which was consumed at a comparable rate that required bolus addition from Day 5 onwards for all cell cultures. Equivalent to the glucose profile, a comparable trend of the lactate concentration during the duration of the cultivation could be observed. Furthermore, analysis of the final titer at the day of the harvest also demonstrated comparable productivity of 0.193, 0.239, 0.235, 0.228 g L^−1^ for On‐demand_1, On‐demand_2, Batch_1, and Batch_2.

Finally, analysis of the essential and non‐essential amino acids at the day of harvest indicated a comparable profile as well (Figure S5).

### 3.5 Product quality

**Figure 9:**
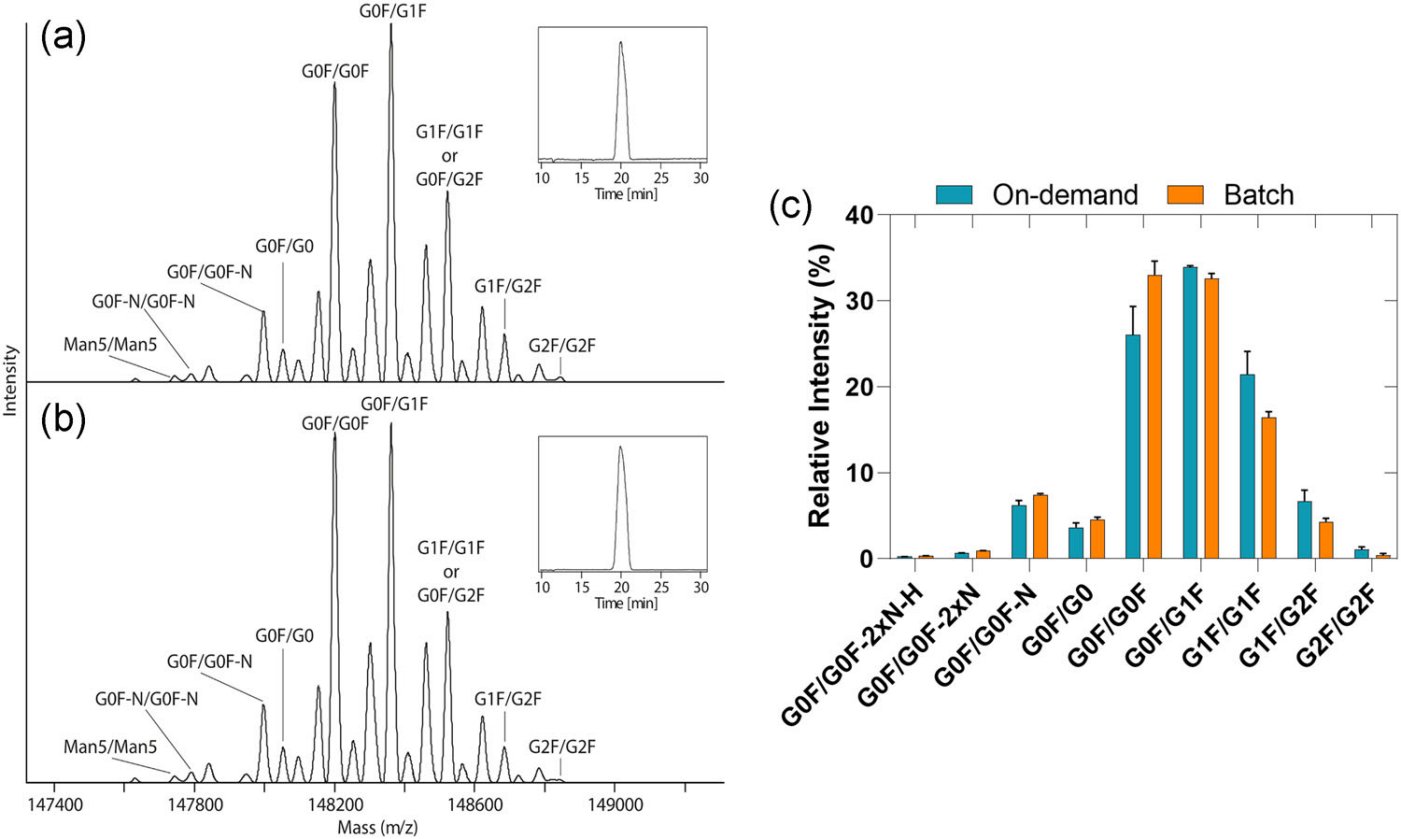
Sheathless CE‐MS of intact mAbs. Deconvoluted mass spectra of (a) continuous on‐demand reconstituted and (b) batchwise reconstituted samples derived from controlled conditions after sheathless CE‐MS separation. The inset shows the base peak electropherograms of both samples. (c) Relative intensity of the glycoforms of mAbs produced with continuous on‐demand reconstituted medium (black) or batchwise reconstituted medium (gray) [Color figure can be viewed at wileyonlinelibrary.com]

The product quality and posttranslational modifications (PTMs) of antibodies obtained with batch and on‐demand reconstituted media were identically measured using sheathless CE‐MS (Figure 9). No additional fragments were observed in the base peak electropherograms (Figure 9). The glycosylation pattern between continuous on‐demand and batchwise reconstitution showed only minor differences that are within the expected batch to batch variations for pharmaceutical mAb production (Planinc et al., 2017; Upton et al., 2019). Importantly, we did not observe significant differences in the levels of afucosylation (Figure 9). The amount of afucosylation is an important CQA because it leads to a high ADCC activation (Ferrara et al., 2006; Li et al., 2017). Regarding other PTMs no differences were observed between the two evaluated conditions.

**Figure 10:**
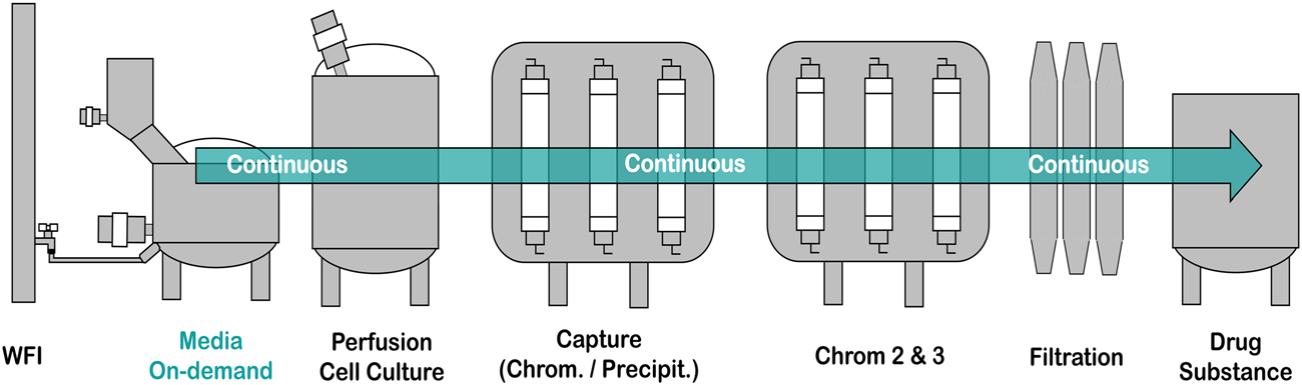
Implementation of a medium‐on demand unit in a process flow scheme proposed by Konstantinov and Cooney (2015) in an integrated continuous bioprocess [Color figure can be viewed at wileyonlinelibrary.com]

Summarizing, using the device for a continuous on‐demand reconstitution, we were able to continuously prepare medium for 12 h with a reconstitution rate of 0.475 L h^−1^. By operating at the lower limits of our miniaturized 3D developed device (19 × 9 × 5 cm), we could theoretically supply continuous on‐demand fresh medium for a 15 L perfusion bioreactor with a working volume of 10 L operating at 1 VVD. In addition, we believe that a nonintegrated stand‐alone version of our developed system similar to already existing automated buffer preparation units could also substantially contribute to the reduction of preparation and storage costs of cell culture media (Carredano et al., 2018; Gibson et al., 2019). The trend of outsourcing media and buffer preparation increased in the last decade, and we believe that an on‐demand reconstitution directly from solids would position itself on the biotherapeutic manufacturing and supplier of process materials side and by that reduce the logistical pressure (Langer, 2016). As the developed device is also easily scalable, we offer a flexible solution for large scale media preparation but also to make continuous processes such as the perfusion technology more easily available for existing fed‐batch facilities (Lin et al., 2017); thus, extending the continuous idea into the auxiliary equipment's of buffer and media preparation (Figure 10).

Furthermore, by following an ICB approach for media reconstitution a new “playground” for media and process development will emerge, considering that the nutrient demand during the growth and production phase (steady‐state) significantly differs (Altamirano et al., 2001; Chen et al., 2021; Kaisermayer et al., 2016; Sieck et al., 2017; Vijayasankaran et al., 2010). By that current solubility and stability issues might be overcome, which could potentially lead to superior decoupled medium formulations for continuous bioprocessing (Salazar, Keusgen, et al., 2016). Also, the development of feeding strategies for perfusion technology such as a gradual increase in nutrients by applying a “specific DPM feeding rate” could introduce new concepts for process control and consequently reduce oscillations in the RT of ICB (Sencar et al., 2020). Thus, a fully automated continuous on‐demand media preparation will contribute to the reduction of floor space, further process intensification but also to flexibility for media development and feeding strategies.

## 4 CONCLUSION

The herein described proof‐of‐concept, showed the possibility to continuously reconstitute CDM directly from solids resulting in the same quality of the medium and consequently also in the same quality of the cell culture product. The long‐term operation over a duration of 12 h demonstrated that such an on‐demand medium product is robust and precise. An on‐demand reconstitution directly from solids will make the repeated preparation of cell culture media and intermediate hold tanks obsolete, which contributes significantly to the reduction of the floor space. By preparing cell culture media directly from solids, new feeding strategies and media formulations can emerge. Limitations by low solubility of compound in concentrated stock solution can be overcome. The developed device will enable media and process development, which will contribute to process efficiency and the implementation of a continuous on‐demand reconstitution of CDM. Transformation of already existing fed‐batch facilities into continuous facilities without increased demand on the auxiliary supply of media and buffers will be possible. The on‐demand direct reconstitution will further advance continuous integrated process and reduce cost and improve environmental impact.

## AUTHOR CONTRIBUTIONS

**Daniel Komuczki**: Constructed the equipment, conducted the experiments, and drafted the manuscript. **Gregory Dutra**: Purified the antibodies and supported them during the long‐term reconstitution. **Christoph Gstöttner**: Performed mass spectrometric analysis, data interpretation, and reviewed the manuscript. **Elena Dominguez‐Vega**: Performed mass spectrometric analysis, data interpretation, and reviewed the manuscript. **Alois Jungbauer**: Wrote the research proposal, drafted the manuscript, and helped with the interpretation of the data. **Peter Satzer**: Wrote the control algorithm, help to design the equipment, converted the design into a 3D print, and reviewed the manuscript.

## Supporting information

Supporting information.
