# Supplementary material for: Media on‐demand: Continuous reconstitution of a chemically defined media directly from solids
Source: Biotechnol Bioeng. 2021 Mar 25;118(9):3382–94. doi: 10.1002/bit.27738 (PMC8451748; doi:10.1002/bit.27738)
Supplement: Supplementary file 1 — Supporting information. [file BIT-118-3382-s001.docx]

**Supporting Information**


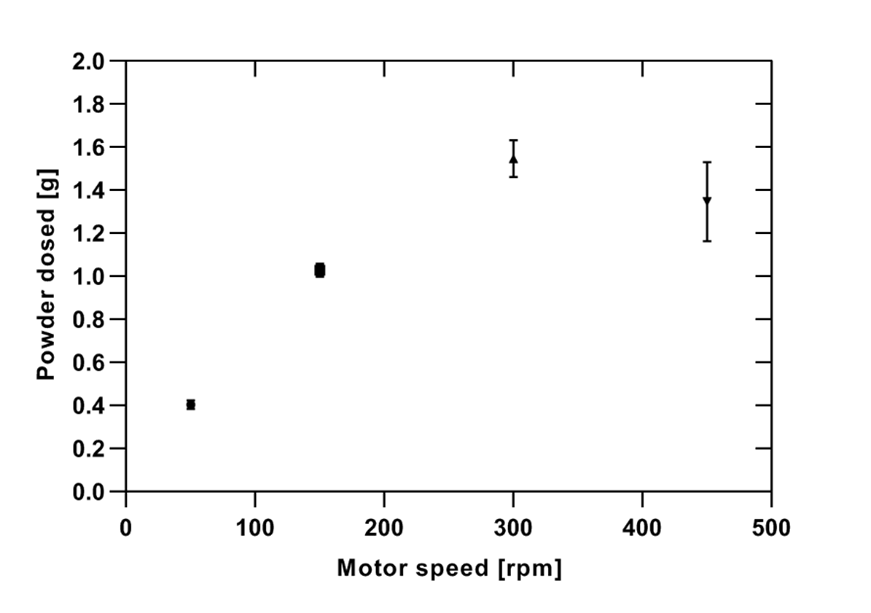


**Figure S1** Calibration curve of the solid media dosing at 50, 150, 300 and 450 rpm. If no error bars can be seen then the error bars are smaller than the data point.


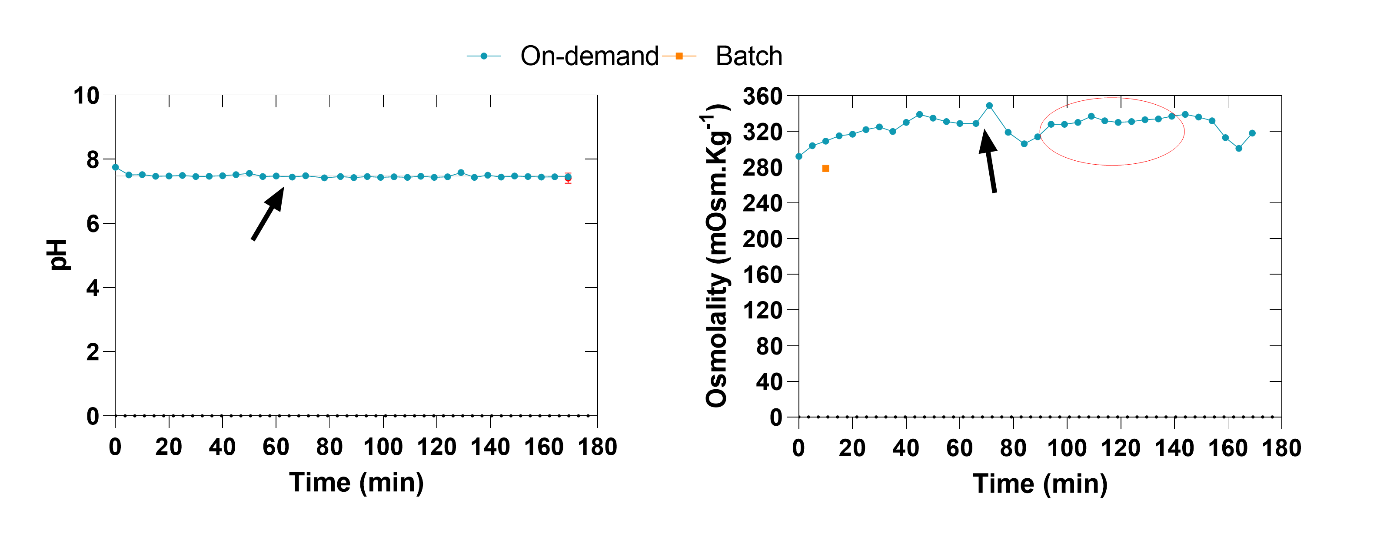


**Figure - S2** pH and Osmolality profiles of a batch and continuously reconstituted basal medium. The

arrow indicates the volume deviation caused by unsynchronized pump feeding rates. The aliquots in the red circle were pooled and used for spin tube experiments.


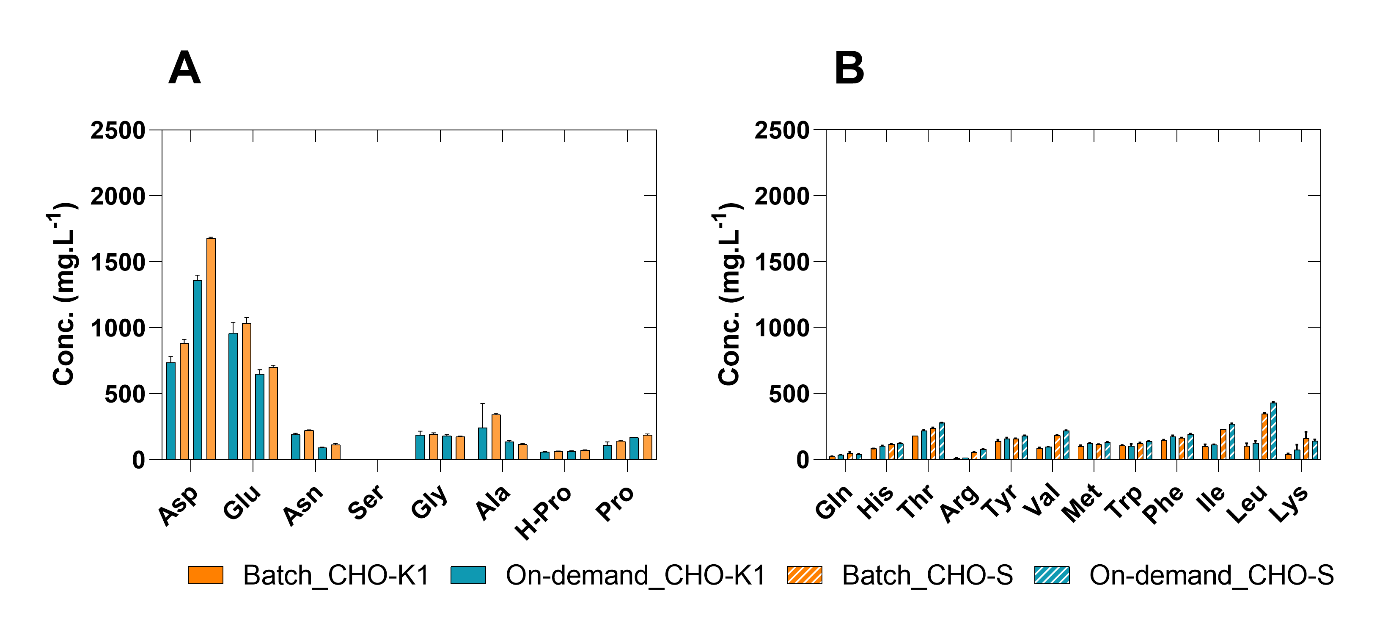


**Figure - S3** The concentration profiles of non-essential (A) and essential (B) amino acids at the end of the cultivation at day 7. Error bars indicate biological quadruplets (n=4) for batch and duplicates (n=2) for continuous on-demand cultures (short-term).





**Figure – S4** Calibration curve and linear regression of the solid media long-term dosin. For illustration purposes the number of data points has been reduced (n = 727).


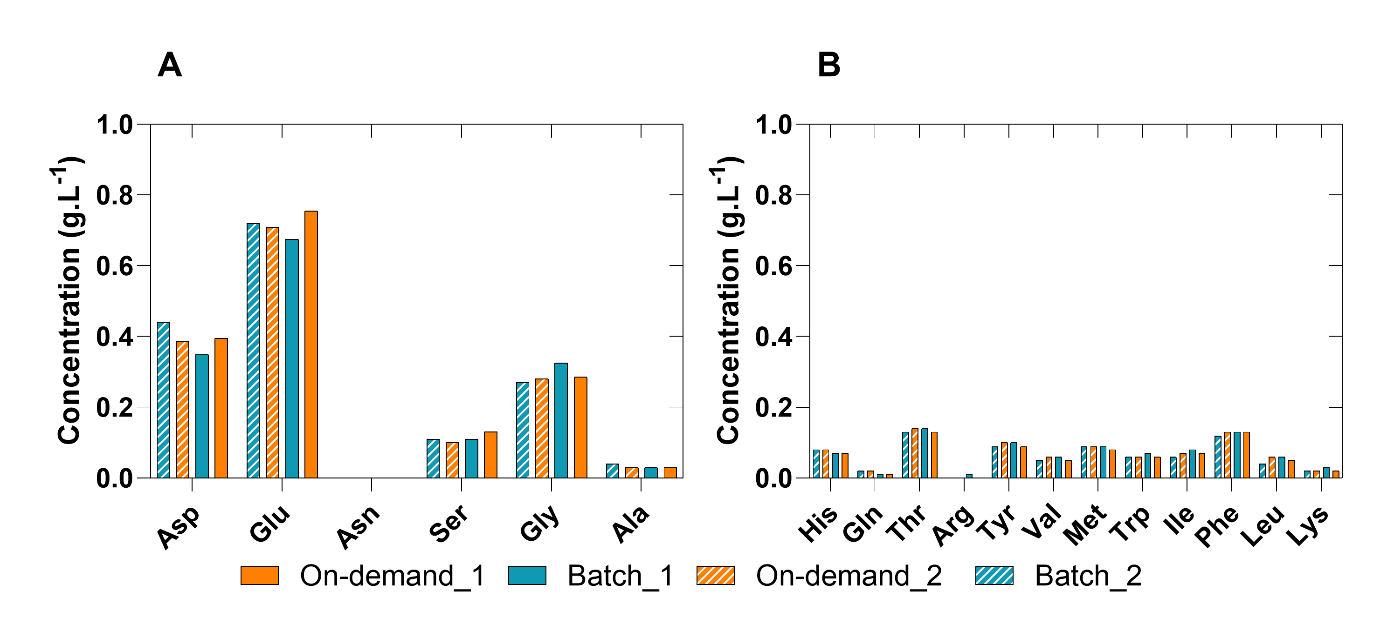
**Figure - S5** AA profiles of non-essential AA (A) and essential AA (B) at the day of harvest of continuously on-demand or batch wise cultured CHO-K1 cells.
